# The effects of residual energy intake on nutrient use, methane emissions and microbial composition in dairy cows

**DOI:** 10.1038/s41598-024-51300-7

**Published:** 2024-01-05

**Authors:** Seppo Ahvenjärvi, Ali-Reza Bayat, Maria Toivanen, Päivi Mäntysaari, Ilma Tapio

**Affiliations:** 1 Natural Resources Institute Finland (Luke), Production Systems, 31600 Jokioinen, Finland; 2 Department of Biology, University of Turku, 20014 Turku, Finland

**Keywords:** Animal physiology, Microbial communities

## Abstract

For sustainable food production selection and breeding of feed efficient animals is crucial. The objective of this study was to evaluate whether multiparous dairy cows, ranked during their first lactation based on residual energy intake (REI) as efficient (low; L-REI) or inefficient (high; H-REI), differ in terms of nutrient use efficiency, methane emissions, rumen fermentation, and gut microbiota composition. Six L-REI and 6 H-REI cows were offered two diets with either a low or high proportion of concentrates (30 vs. 50% of DM) on two consecutive periods of 21 d. Gas exchanges, milk yield, feces and urine excretions were measured in open-circuit respiratory chambers. The results indicated that L-REI cows had higher methane yields (22.6 vs. 20.4 g/kg DM intake) and derived more energy (energy balance − 36.6 vs. − 16.9 MJ/d) and protein (N balance − 6.6 vs. 18.8 g/d) from the tissues to support similar milk yields compared to H-REI cows. Nutrient intake and digestibility were not affected by REI, and there were no interactions between REI and diet. Milk yield, milk production efficiency, and milk composition were not affected by REI except for milk urea concentration that was higher for L-REI cows (14.1 vs. 10.8 mg/100 ml). The rumen and fecal microbiota community structure and function were associated with both the diet and REI, but the diet effect was more pronounced. The current study identified several physiological mechanisms underlying the differences between high and low REI cows, but further studies are needed to distinguish the quantitative role of each mechanism.

## Introduction

The world’s growing population requires increasing amounts of food, while any expansion in forest clearance for agricultural land use should be avoided. Currently, approximately 37% of global land area is cleared for agricultural use^1^, but the amount of arable land per person will decrease in the future^2^. Livestock production accounts for a large share of global land resources and the environmental footprint of the human population^3^. Consumption of animal-based foods has been criticized for low biomass transfer efficiency compared to consumption of vegan or vegetarian diets^4^. This is a valid but narrow aspect of the role of ruminants in human food production systems. Ruminants are needed to convert human nonedible feed resources to nutrient-dense food, which in combination with plant-based foods provides well-balanced diets, especially for growing and elderly people^5^. Therefore, to provide sustainable choices for human diets, the efficiency of milk and beef production must be improved in terms of nutrient use efficiency and conversion of feed resources to human-edible food^6^.

The milk yield and milk production efficiency of dairy cows have increased substantially over the last half-century. For instance, in North America, the feed efficiency of dairy cows has increased by 100% over the last 50 years because of increased milk production per cow attained through genetic selection as well as advances in nutrition and management^7^. In Finland, milk yield per cow has increased by approximately 300%, the amount of feed required to produce 1 kg/d of energy corrected milk (ECM) has decreased from 1.23 to 0.82 kg/d of dry matter (DM), and the methane (CH_4_) intensity of milk production (g CH_4_ per kg ECM) has decreased by 36% between 1960 and 2020^8^. Recently, feed efficiency has gained more attention in genetic selection because the environmental footprint of animal products has been the focus of debate. To gain progress in feed efficiency, residual feed intake (RFI) defined as the difference between measured and predicted DM intake or residual energy intake (REI) defined as the difference between measured and predicted energy intake have been suggested as phenotypic traits that should be incorporated into selection programs to decrease feed input per unit of milk yield^13^. A crucial assumption in RFI measurements is that between-cow variation that is not accounted for by the energy sinks (ECM yield, metabolic body weight (BW), and BW change) can be attributed to differences in energy use efficiency^9^.

Feed efficiency is a complex trait. Underlying variation in feed efficiency phenotypes among animals has been associated with ruminant gut microbial community structure and function^10,11^. This relationship is not surprising, considering that up to 70% of the energy available for the animal is produced through microbial fermentation and digestion of consumed feeds in the rumen^12^. If the feed efficiency phenotype is to be used for breeding purposes, the key questions that remain open due to inconsistent findings are the persistence of the efficiency phenotype across lactations and different diets and the usability of the gut microbiota as a biomarker for defining feed efficiency.

For this study, we recruited multiparous dairy cows that have been followed up for their efficiency during their whole 1st lactation period. The objective of the current study was to evaluate if these multiparous dairy cows ranked earlier as efficient (low; L-REI) and inefficient (high; H-REI) cows will demonstrate differences in terms of nutrient use efficiency, rumen fermentation, methane emissions, and rumen or fecal microbiota composition. To test this hypothesis, dairy cows were offered two diets with different energy densities manipulated by two levels of concentrate inclusion in the diet.

## Methods

### Animals, experimental design, and diets

All experimental procedures were approved by the Project Authorisation Board (Regional Administrative Agency for Southern Finland, Hämeenlinna, Finland; ESAVI/17310/2021) in accordance with the guidelines established by the European Community Council Directive 86/609/EEC and in compliance with ARRIVE guidelines. Twelve multiparous (mean ± SD; 3.6 ± 1.2 parturitions) Nordic Red dairy cows were selected from the cohort of approximately 120 dairy cows in Luke research barn based on calving date and their earlier ranking with respect to REI determined during their first lactation. Residual energy intake was calculated from milk yield, feed intake, and body weight data collected throughout lactation by fitting a multiple linear regression model with ECM output, metabolic body weight (BW^0.75^), and piecewise regressions of body weight gain or body weight loss on the total metabolizable energy (ME) intake, as described in detail in^13^. From the cows that were calving around the same time, most divergent animals in terms of REI were selected for the experiment. Six multiparous dairy cows which had negative REI (− 9.5 ± 2.4 MJ/d) in their 1st lactation were assigned to the group of cows named as low REI (L-REI), and 6 cows with earlier positive REI (10.7 ± 3.7 MJ/d) were assigned to the group named as high REI (H-REI). Cows with 49 ± 11.2 days in milk (DIM) and BW 649 ± 47.4 kg at the beginning of the study were assigned to a replicated change-over study with two experimental treatments and two 21-d periods. The cows in the L-REI group were slightly heavier (661 ± 54.5 kg) than those in the H-REI group (637 ± 40.4 kg). The cows were housed in a free-stall barn and had constant access to water and salt blocks throughout the study. To allow gas exchange measurements in four open-circuit respiratory chambers, cows were allocated to 3 squares of 4 cows according to date of parturition and REI. Two H-REI and 2 L-REI cows were allocated to each square. Each square started the experiment with one-week intervals. In each period, cows were allowed to adapt to changes in the diet for 16 d. On d 17, cows entered the respiration chambers, and on d 18 to 21, measurements were carried out.

Treatments consisted of two diets based on grass silage supplemented with either a low (30% of DM, diet LC) or high (50% of DM, diet HC) proportion of concentrates. While the HC diet was typical for dairy cows on commercial farms in Finland, the LC diet was chosen as an alternative to represent diets based on high-quality forages with moderate to low proportions of concentrates in response to increasing public demand for sustainable food systems. Grass silage was prepared from timothy-meadow fescue sward that was cut using a mower conditioner, allowed to slightly wilt, and then harvested using a precision chopper. Formic and propionic acid‒based additive (AIV Ässä Na, Eastman/Taminco Finland Oy, Oulu, Finland) was applied at harvest at a rate of 5 L/ton and preserved in a clamp until the beginning of the experiment. One batch of concentrate ingredients was mixed and pelleted for the entire experiment. The concentrate mixture consisted of (g/kg as fed) barley 243, oats 243, sugar beet pulp 243, solvent extracted rapeseed meal 240, and a mixture of minerals and vitamins 31. The grass silage and concentrate mixture prepared as total mixed ration (TMR) was offered ad libitum four times daily at 0700, 1300, 1615 and 1800 h, allowing no less than 10% for the refusals. In the chambers, TMR was fed at 0600, 1130, 1630 and 1930 h. Cows received 0.53 kg DM concentrate per day in the milking parlor twice daily. The chemical composition of the experimental feeds and diets is presented in Table 1.

**Table 1:** Chemical composition of feed ingredients and diets.

	Grass silage^1^	Milking parlor concentrate^2^	Concentrate mixture^3^	Diet LC^4^	Diet HC^5^
DM, g/kg	230	876	878	299	366
Composition, g/kg DM					
OM	934	923	917	929	926
Digestible OM^6^	706	NA^8^	NA	NA	NA
CP	121	188	184	140	152
Ether extract	35	25	35	35	35
Starch	NA	360	263	81	132
NDF	522	209	259	441	390
GE, MJ/kg DM^7^	18.3	18.2	18.4	18.3	18.3

^1^Fermentation quality: pH 3.9; In DM, g/kg: lactic acid 52, acetic acid 20, propionic acid 0.3, butyric acid 0.2, ethanol 9, water soluble carbohydrates 21; In total N, g/kg: soluble N 452, ammonium-N 39.

^2^Parlor concentrate (0.53 kg DM/d) was offered twice daily in the milking parlor.

^3^Pelleted mixture of concentrate ingredients was mixed with grass silage as TMR.

^4^Diet LC consisted of a low (30% of DM) proportion of concentrates in the total mixed ration.

^5^Diet HC consisted of a high (50% of DM) proportion of concentrates in the total mixed ration.

^6^Concentration of digestible OM based on cellulase solubility in vitro^21^.

^7^Gross energy.

^8^Not analyzed.

### Sample collection and measurements

Samples of grass silage were collected two times a week for the analysis of DM. To determine the chemical composition of grass silage and concentrates, samples were collected daily on d 17–20 of each experimental period. Milk yield was measured daily throughout the experiment, but those recorded on d 17–20 are reported. For the analysis of milk composition, milk samples of 30 mL were collected from six consecutive milkings on d 18–d 21. Samples were preserved with bronopol and submitted to a commercial lab (Valio Oy, Seinäjoki, Finland) for infrared analysis (MilkoScan FT+, Foss Electric) of fat, crude protein, lactose, and urea concentrations and somatic cell count. The live weight of each cow was recorded on two consecutive days at the beginning of each period and at the end of the study.

Exchanges of oxygen, carbon dioxide, and methane were measured over 4 d using 4 open-circuit respiratory chambers (21.5 m^3^). The details of the gas exchange measurements have been described previously^14^. In brief, cows entered the chambers for 4 d on d 17 of each period. The first day was assigned to adaptation to chamber conditions, and the exchange of gases was measured on days 18–21. Concentrations of gases in the inlet and exhaust air flow were measured using a computer-controlled system equipped with dedicated analyzers (Oxymax, Columbus Instruments, Columbus, OH, USA). Air outflow from each chamber was measured using an HFM-200 mass flow meter with a laminar flow element capable of measuring up to 3000 L/min (Teledyne Hastings Instruments, Hampton, VA, USA). Absolute gas exchanges corrected for standard temperature and pressure (STP) conditions were calculated based on air flow and gas concentration differences. Before running the main experiment, gas recovery tests were conducted. Nominal amounts of CO_2_ (3.5, 5.0, 6.5 and 8.0 L/m equal to 5040, 7200, 9360 and 12,240 L/d) and CH_4_ (0.2, 0.4, or 0.6 L/m equal to 288, 576 and 884 L/d) were set by flow meters (AGA, Espoo, Finland) and were released into every individual respiratory chamber for at least 2 h. The gas levels were selected to represent the wide range of CO_2_ and CH_4_ production by the cows based on previous experiments. The data recorded after achieving a plateau in the outlet air CO_2_ and CH_4_ concentrations (after approximately 1 h from the initiation of gas release) were used to calculate the gas recovery ratio. For a more accurate measurement of released gas, the gas cylinders were weighed by scale (20 kg ± 1.0 g) before and after releasing each gas, and the actual gas release rate was calculated. For O_2_, a different approach was taken, and CO_2_ injection effects were considered as a dilutor for O_2_. The measured versus released amounts of gases were used to calculate whole system gas recovery (measured gas/released gas × 100). The oxygen, CO_2_, and methane recoveries were 100.1 ± 0.04, 103.1 ± 5.8, and 100.7 ± 1.45% (mean ± SD), respectively. The heat production of the cows was calculated according to the following equation^15^:

$${\text{Heat }}\left( {{\text{kJ}}/{\text{d}}} \right) = {16}.{18} \times {\text{O}}_{{2}} + {5}.0{2} \times {\text{CO}}_{{2}} - {2}.{17} \times {\text{CH}}_{{4}} - {5}.{99} \times {\text{N}},$$

where O_2_, CO_2_ and CH_4_ exchanges are expressed as L/d and N is the urinary N as g/d. In the chambers, feces and urine were collected over 3 d to determine nutrient digestibility and utilization. The total digestive tract apparent digestibility of DM and nutrients was calculated as the difference between their intake and excretion in feces divided by the intake. Urine was drained into containers using harnesses, and its pH was adjusted below 3 using 10 *N* H_2_SO_4_. Feces were collected into trays placed below slatted floor level. The amount of urine and feces excreted over 12 h was weighed, and representative samples were obtained and stored frozen at − 20 °C until analysis of the chemical composition. Energy balance was calculated as the difference between energy intake and energy excreted as feces, urine, milk, methane, and heat.

On d 21 at 1000 h of each experimental period immediately after the cows left the chambers, samples of rumen liquid (500 mL) were collected via the esophagus using a Ruminator device (Profs Products, Wittibreut, Germany) to determine rumen VFA concentrations and rumen microbiome composition. In case of saliva presence, rumen liquid was discarded, and new sample (500 mL) was collected. Immediately after collection, rumen liquid was strained, and a subsample of 5 mL was obtained, mixed in a plastic vial with 0.5 mL of saturated mercuric chloride and 2.0 mL of 1 M NaOH solutions, chilled on ice and stored at − 20 °C. For rumen microbial analysis, rumen liquid samples of 2 mL were added to screw cap tubes, and the contents were snap frozen in dry ice and stored at − 80 °C until DNA extraction.

### Chemical analysis

Samples of feed ingredients, feces and urine were stored at − 20 °C until analyzed for DM and prepared for chemical composition. Feed and fecal samples were dried in a forced-air oven at 60 °C and milled for chemical analysis using a 1-mm screen. The dry matter concentration of milled samples was determined based on weight loss in a forced-air oven at 105 °C for 16 h. Preparation of samples has been described in detail by Ahvenjärvi et al.^16^. The grass silage DM concentration was corrected for the loss of volatile compounds as described by Huida et al.^17^. The ash concentration was analyzed according to official method AOAC-942.05. The nitrogen concentration in fresh samples of feces and urine was determined by the Kjeldahl method using CuSO_4_ as a catalyst. The nitrogen concentration in dry feed samples was determined using a Dumas-type elemental N analyzer (Leco FP-428, Leco Corporation, St. Joseph, MI, USA). Starch concentrations in feed ingredients were determined according to Salo and Salmi^18^. The neutral detergent fiber (NDF) concentration was analyzed according to Van Soest et al.^19^ in the presence of Na_2_SO_3_ using an Ankom 220 fiber analyzer (Ankom Technology, Macedon, NY, USA). Heat-stable α-amylase was used for concentrate samples. The ether extract concentration in feed ingredients was determined according to AOAC Official Method 920.39 and in feces after hydrolysis with 3 M HCl. Gross energy (GE) was determined using a Parr 6200 oxygen bomb calorimeter (Parr Instrument Co., Moline, IL, USA) with benzoic acid as a standard. Digestibility of grass silage organic matter (OM) was determined based on pepsin-cellulase digestion in vitro according to Nousiainen et al.^20^ incorporating the modification described by Huhtanen et al.^21^. The concentration of digestible OM in grass silage was estimated using the equation suggested by^21^: digestible OM (g/kg of DM) = [1000 – ash (g/kg of DM)] × [0.077 + 0.86 × OM solubility (g/g of OM)]. Volatile fatty acid concentrations in grass silage and rumen fluid were determined by gas chromatography as described in^22^.

### Ruminal and fecal microbial analysis

Total DNA was extracted from 0.5 mL of rumen liquid and ca. 245 mg of feces following the protocol described by Rius et al.^23^. Rumen and fecal prokaryotic community composition was determined using universal primers 515F and 806R^24^ for 16S rRNA gene V4 region amplicon sequencing. The sequencing library was prepared as described by Huuki et al.^25^ and sequenced at the Finnish Functional Genomics Centre (Turku, Finland) on an Illumina MiSeq platform by using 2 × 300 bp chemistry. The sequencing data are available in the NCBI Sequence Read Archive under BioProject Accession PRJNA922943. Demultiplexing of sequences, adapter removal, and sorting sequences by barcode were performed by the sequencing center. Sequence read quality control that included filtering, denoising, merging and removal of chimeric reads as well as clustering of bacterial sequences into amplicon sequence variants (ASV) at 99% similarity was performed using DADA2^26^ following the default settings in QIIME 2^27^. From 61,000–131,000 raw sequences per rumen sample, 18,792–40,248 reads per sample passed the quality control. For fecal samples 53,000–128,000 raw sequencing reads per sample were generated and 16,244–36,869 remained after quality filtering. ASVs with less than 10 reads in total were removed. Bacterial ASV taxonomy was assigned using the Silva 138 database^28^. After filtering out bacterial sequences, we retained 150–521 archaeal reads per sample for rumen and 40–527 reads for feces and assigned archaeal taxonomy using the RIM-DB database^29^.

### Statistical analysis

Statistical analyses were carried out using PROC GLIMMIX of SAS 9.4 software (SAS Institute Inc., Cary, NC, USA). The observations were analyzed using the following model:

$${\text{Y}}_{{{\text{ijklm}}}} = \mu + {\text{a}}_{{\text{i}}} + {\text{s}}_{{\text{j}}} + {\text{P}}_{{\text{k}}} + {\text{R}}_{{\text{l}}} + {\text{D}}_{{\text{m}}} + {\text{RD}}_{{{\text{lm}}}} + \varepsilon_{{{\text{ijklm}}}} ,$$

where µ is the intercept; a_i_ (_i_ = 1–12) and s_j_ (_j_ = 1–3) represent the random effects of animal and square; P_k_, R_l_, and D_m_ represent the fixed effects of period (_k_ = 1–4), REI (_l_ = 1–2), and diet (_m_ = 1–2), respectively; RD_lm_ represents the interaction between REI and diet, and ε_ijklm_ represents residual variation. The degrees of freedom were calculated using the Kenward-Roger method.

The bacterial community alpha diversity of ruminal and fecal samples was estimated using the Shannon diversity index and richness (number of observed ASVs) as implemented in the *MicrobiotaProcess* R package^30^. The data were evenly subsampled to the lowest number of reads per sample in each data set, and significant differences in pairwise comparisons were estimated using the nonparametric Wilcoxon test. To evaluate diet and REI effects on the changes in ruminal and fecal microbial community structure, between-sample diversity was calculated as Bray–Curtis dissimilarities following Hellinger transformation and visualized using principal coordinate analysis (PCoA) and boxplots. The significance of groups was evaluated by distance-based permutational multivariate analysis of variance (adonis) and defined at the *P* < 0.05 level after 999 permutations, as implemented in the *vegan* R package^31^. Diet and REI effects on individual microbial genera were evaluated using PROC GLIMMIX as described above. Before the analysis, all genera below 0.01% abundance in more than 50% of samples were filtered out, and the number of reads was log base transformed [log_2_ (x + 1)] and standardized by data centering. For easier interpretation of the results, microbial genera significantly affected by diet, REI or the interaction between REI and diet were converted back to compositional data and presented in Table 7 as relative abundances. Prediction of functional profiles from 16S rRNA gene amplicon data was performed using CowPI^32^. The ASV table was imported into CowPI, and the Kyoto Encyclopedia of Genes and Genomes database was used to predict the functional gene content of the microbial communities, comparing them against the rumen-curated database created from Global Rumen Census and Hungate 1000 data collections. The differences in mean proportions of the predicted functional categories, clustered by diet and/or REI, were evaluated using STAMP v2.1.3^33^.

## Results

### Nutrient intake, digestibility, and milk yield

A high proportion of concentrates in the diet decreased (*P* < 0.01) grass silage DM intake from 15.9 to 12.9 kg/d and increased (*P* < 0.01) concentrate DM intake from 6.9 to 12.7 kg/d and that of total DM from 22.8 to 25.6 kg/d (*P* < 0.01). A high proportion of concentrates increased (*P* < 0.01) the daily intake of OM, crude protein (CP), ether extract, and starch (Table 2). Feed intake was not affected by REI, and there were no interactions between REI and diet in feed intake (Table 2). Nutrient digestibility was not affected by the diet or REI except for NDF and ether extract digestibility that decreased in response to a higher proportion of concentrates.

**Table 2:** The effects of residual energy intake (REI) and diet on nutrient intake and digestibility.

Diet	H-REI^1^		L-REI^2^			*P* values		
	LC^3^	HC^4^	LC	HC	SEM	REI	Diet	REI × Diet
Intake, kg/d								
Silage DM	16.1	13.3	15.7	12.6	0.67	0.53	< 0.01	0.59
Concentrate DM	6.9	13.1	6.8	12.3	0.49	0.35	< 0.01	0.20
DM	23.0	26.4	22.5	24.9	1.14	0.45	< 0.01	0.28
OM	21.4	24.5	20.9	23.0	1.05	0.45	< 0.01	0.28
CP	3.22	4.01	3.14	3.78	0.151	0.46	< 0.01	0.33
NDF	10.16	10.30	9.93	9.71	0.48	0.48	0.83	0.33
Ether extract	0.82	0.92	0.78	0.86	0.033	0.41	< 0.01	0.30
Starch	1.87	3.53	1.84	3.25	0.147	0.28	< 0.01	0.16
OM digested	14.9	17.1	14.8	15.9	0.82	0.43	< 0.01	0.27
Gross energy, MJ/d	421	484	412	455	20.3	0.45	< 0.01	0.29
Digestibility, %								
OM	69.5	70.1	70.5	69.2	1.01	0.97	0.71	0.40
CP	62.8	64.5	62.6	62.5	1.09	0.33	0.45	0.41
NDF	59.0	54.4	61.2	54.4	1.62	0.55	< 0.01	0.50
Ether extract	56.5	60.0	56.0	59.0	1.26	0.58	0.03	0.84
Gross energy	65.7	67.2	66.9	66.1	1.09	0.97	0.77	0.32

^1^High REI group of cows.

^2^Low REI group of cows.

^3^Containing a low (30% DM) proportion of concentrates in the total mixed ration.

^4^Containing a high (50% DM) proportion of concentrates in the total mixed ration.

Milk, ECM, and milk component yields were higher (*P* < 0.01) for HC diets than for LC diets (Table 3). Milk composition was not affected by the diet except for urea concentrations that were higher (*P* = 0.01) for the HC diets. Milk urea concentration was higher (*P* = 0.01) for L-REI cows than for H-REI cows. Milk production efficiency was not affected by the diet or REI.

**Table 3:** The effects of residual energy intake (REI) and diet on milk yield and composition.

Diet	H-REI^1^		L-REI^2^			*P* values		
	LC^3^	HC^4^	LC	HC	SEM	REI	Diet	REI × Diet
Yield, kg/d								
Milk	32.5	38.2	33.2	39.3	1.39	0.63	< 0.01	0.77
ECM^5^	35.6	41.3	35.7	41.9	1.39	0.87	< 0.01	0.77
Fat	1.55	1.77	1.54	1.76	0.073	0.90	< 0.01	1.00
Protein	1.14	1.33	1.12	1.37	0.049	0.91	< 0.01	0.28
Lactose	1.47	1.75	1.53	1.83	0.064	0.40	< 0.01	0.79
Total solids	4.50	5.24	4.53	5.35	0.170	0.76	< 0.01	0.68
Milk composition, %								
Fat	4.78	4.66	4.62	4.48	0.179	0.49	0.19	0.93
Protein	3.52	3.50	3.38	3.48	0.088	0.22	0.57	0.39
Lactose	4.52	4.57	4.61	4.65	0.048	0.25	0.10	0.88
Total solids	13.9	13.8	13.6	13.6	0.23	0.53	0.55	0.67
Urea, mg/100 ml	8.5	13.0	12.3	15.8	1.42	0.01	0.01	0.65
Milk production efficiency								
ECM/OM intake, kg/kg	1.67	1.71	1.71	1.83	0.101	0.15	0.12	0.40
ECM/OM digested, kg/kg	2.41	2.44	2.43	2.66	0.165	0.12	0.11	0.22
ECM/GE^6^ intake, kg/MJ	0.0849	0.0863	0.0869	0.0924	0.0050	0.15	0.16	0.42

^1^High REI group of cows.

^2^Low REI group of cows.

^3^Containing a low (30% DM) proportion of concentrates in the total mixed ration.

^4^Containing a high (50% DM) proportion of concentrates in the total mixed ration.

^5^Energy corrected milk calculated according to Sjaunja et al. (1990).

^6^Gross energy.

### Rumen fermentation and methane emissions

Rumen pH was lower (*P* < 0.05) and rumen total VFA concentrations were higher (*P* = 0.05) for L-REI cows than for H-REI cows (Table 4). The rumen molar proportions of VFA were not different between L-REI and H-REI cows but were affected by diet. Molar proportions of acetate and isobutyrate decreased (*P* < 0.05) and those of propionate, valerate, and caproate increased (*P* < 0.01) as a response to a high proportion of concentrates in the diet. These changes in rumen fermentation were accompanied by decreases (*P* < 0.01) in the acetate to propionate ratio for the HC diet.

**Table 4:** The effects of residual energy intake (REI) and diet on rumen fermentation.

Diet	H-REI^1^		L-REI^2^			*P* values		
	LC^3^	HC^4^	LC	HC	SEM	REI	Diet	REI × Diet
pH	6.77	6.76	6.57	6.57	0.069	0.03	0.96	0.96
Total VFA, m*M*	97	97	104	111	5.7	0.05	0.36	0.36
Molar proportions, mmol/mol								
Acetate	689	666	684	669	4.7	0.90	< 0.01	0.18
Propionate	157	175	159	172	3.8	0.90	< 0.01	0.39
Butyrate	124	127	126	127	2.5	0.84	0.35	0.40
Isobutyrate	6.35	5.69	6.16	5.79	0.222	0.81	0.04	0.54
Valerate	11.5	13.1	11.2	12.8	0.34	0.58	< 0.01	0.76
Isovalerate	6.41	5.78	7.66	7.19	0.851	0.19	0.17	0.84
Caproate	5.43	6.70	5.38	6.18	0.305	0.32	< 0.01	0.43
Acetate: Propionate	4.42	3.81	4.33	3.90	0.126	0.99	< 0.01	0.33

^1^High REI group of cows.

^2^Low REI group of cows.

^3^Containing a low (30% DM) proportion of concentrates in the total mixed ration.

^4^Containing a high (50% DM) proportion of concentrates in the total mixed ration.

A high proportion of concentrates increased (*P* < 0.01) methane emissions, but these effects were associated with higher DM intake, as methane yield (g/kg of DM intake) tended to decrease (*P* = 0.06) with HC diet. Methane intensity (g/kg of milk or ECM yield) decreased significantly (*P* < 0.01) for HC than LC diet (Table 5). Methane yield and methane emissions per unit of OM digested in the total tract were higher (*P* < 0.01) for L-REI than H-REI cows, but this was not the case with methane intensities. There were no interactions between REI and diet in terms of methane emissions, yield or intensities. Carbon dioxide emissions were higher (*P* < 0.01) for the HC diet than for the LC diet, but the CO_2_ yield (g/kg DMI) was not different between the diets. On the other hand, CO_2_ emissions were not different between efficiency groups, but L-REI had a higher (*P* < 0.01) CO_2_ yield than H-REI. Oxygen consumption was not different between the REI groups, but it was higher (*P* < 0.01) for the HC diets than for the LC diets.

**Table 5:** The effects of residual energy intake (REI) and diet on gas exchanges.

Diet	H-REI^1^		L-REI^2^			*P* values		
	LC^3^	HC^4^	LC	HC	SEM	REI	Diet	REI × Diet
Methane								
g/d	488	516	511	559	22.1	0.30	< 0.01	0.23
g/kg DM intake	21.2	19.6	22.7	22.5	0.48	< 0.01	0.06	0.15
g/kg OM digested	32.9	30.2	34.7	35.3	1.04	< 0.01	0.32	0.14
g/kg Milk	15.1	13.6	15.4	14.3	0.78	0.49	< 0.01	0.42
g/kg ECM	13.7	12.5	14.4	13.4	0.66	0.18	< 0.01	0.70
% of gross energy intake	6.46	5.95	6.92	6.84	0.145	< 0.01	0.06	0.17
CO_2_								
g/d	14,159	15,364	14,671	16,059	542.0	0.44	< 0.01	0.54
g/kg DM intake	616	584	654	648	18.7	< 0.01	0.11	0.29
g/kg OM digested	953	901	999	1017	40.6	0.02	0.54	0.25
g/kg Milk	438	404	443	409	16.4	0.77	< 0.01	0.97
g/kg ECM	398	373	413	384	14.1	0.37	< 0.01	0.79
O_2,_ g/d	9666	10,490	9929	10,874	386	0.56	< 0.01	0.56
H_2_, g/d	0.49	1.05	0.44	0.89	0.106	0.34	< 0.01	0.48

^1^High REI group of cows.

^2^Low REI group of cows.

^3^Containing a low (30% DM) proportion of concentrates in the total mixed ration.

^4^Containing a high (50% DM) proportion of concentrates in the total mixed ration.

### Energy and N metabolism

A high proportion of concentrates in the diet increased (*P* < 0.01) GE and ME intakes, decreased (*P* < 0.01) the proportion of GE intake lost in urine, and tended to decrease (*P* = 0.06) the proportion of GE lost in methane (Table 6). Cows in the L-REI group lost a higher proportion of GE intake in methane. Higher proportional losses in heat energy (*P* < 0.01) were related to numerically lower GE intake (434 vs. 454 MJ/d, *P* = 0.45) and numerically higher heat production (154 vs. 149 MJ/d, *P* = 0.54) for L-REI cows than for H-REI cows. Energy balance was negative for both diets and REI groups, but it was lower (*P* < 0.05) for L-REI than H-REI cows, and the proportion of milk energy of ME intake tended to be higher (*P* = 0.08) for L-REI cows. No interactions were significant between REI and diet in energy metabolism, but there was a tendency (*P* = 0.12) for interactions between diet and REI group, such that energy balance numerically increased in H-REI cows but decreased in L-REI cows as a response to an increase in concentrate proportion.

**Table 6:** The effects of residual energy intake (REI) and diet on energy and N metabolism.

Diet	H-REI^1^		L-REI^2^			*P* values		
	LC^3^	HC^4^	LC	HC	SEM	REI	Diet	REI × Diet
Energy								
GE intake, MJ/d^5^	421	484	412	455	20.3	0.45	< 0.01	0.29
ME intake, MJ/d	230	277	226	252	13.3	0.27	< 0.01	0.25
Proportion of GE intake, KJ/MJ								
Feces	343	328	331	339	10.9	0.97	0.77	0.32
Urine	47.6	39.8	50.1	39.8	1.70	0.49	< 0.01	0.46
Methane	64.6	59.5	69.2	68.4	1.45	< 0.01	0.060	0.17
Milk	267	271	273	290	15.7	0.15	0.16	0.42
Heat	339	322	358	354	10.1	< 0.01	0.13	0.34
RQ^6^	1.08	1.07	1.07	1.06	0.006	0.23	0.92	0.96
Milk energy/ME intake, KJ/MJ	489	473	497	530	34.4	0.08	0.61	0.19
Energy balance, MJ/d	− 26.1	− 7.6	− 31.5	− 41.6	11.21	0.03	0.60	0.12
Nitrogen (N)								
N intake, g/d	515	641	503	605	24.1	0.46	< 0.01	0.33
Proportion of N intake, g/kg								
Feces	372	355	374	375	10.8	0.33	0.45	0.41
Urine	279	260	288	284	5.4	0.02	0.04	0.14
Milk	349	328	350	355	15.3	0.06	0.26	0.10
N balance, g/d	0.7	36.9	− 5.6	− 7.5	14.0	0.03	0.11	0.10

^1^High REI group of cows.

^2^Low REI group of cows.

^3^Containing a low (30% DM) proportion of concentrates in the total mixed ration.

^4^Containing a high (50% DM) proportion of concentrates in the total mixed ration.

^5^Gross energy.

^6^Respiration quotient (CO_2_/O_2_, on volume basis).

A high proportion of concentrates in the diet increased (*P* < 0.01) N intake but decreased (*P* < 0.05) the proportion of N excreted in urine (Table 6). Cows in the L-REI group excreted a higher proportion of N in urine (*P* < 0.05) and milk (*P* = 0.06), but their N balance was negative (− 7 g/d), whereas that was positive for H-REI cows (19 g/d). Cows in the L-REI group tended to respond more positively to increases in concentrate proportion in terms of milk N synthesis as a proportion of N intake (interaction between REI and diet *P* = 0.10).

### Rumen microbiota

Diet had no significant influence on bacterial alpha diversity in L-REI cows, but H-REI cows fed the HC diet exhibited significantly higher (*P* < 0.05) Shannon diversity and richness than H-REI cows fed the LC diet or L-REI cows receiving the same (HC) diet. Rumen archaeal alpha diversity tended to be higher (*P* = 0.053) in H-REI cows than in L-REI cows when the HC diet was offered (Supplementary Figure S1).

Across the whole data set, ruminal bacteria were dominated by Bacteroidota and Firmicutes, with Proteobacteria, Patescibacteria, Verrucomicrobiota, Fibrobacterota, Actinobacteriota and Spirochaetota, among others, representing low abundance phyla. The ten most abundant genera that made up to 50% of all sequencing reads were affiliated with *Prevotella*, *Prevotellaceae* UCG-001, *Rikenellaceae* RC9 gut group, *Clostridia* UCG-014, *Lachnospiraceae* NK3A20 group, *Oscillospiraceae* NK4A214 group, *Ruminococcus*, *Christensenellaceae* R-7 group, *Bacteroidales* F082 and *Muribaculaceae*. The archaeal community was dominated by *Methanobrevibacter ruminantium* and *Mbb*. *gottschalkii* clades followed by *Methanosphaera ISO3-F5*, with *Methanomassillicoccaceae* groups 10, 8, 12, 9 and 3b detected at lower abundances (Supplementary Figure S2).

Diet and REI effects on rumen microbial community structure were evaluated by comparing differences in Bray‒Curtis (BC) dissimilarities and visualizing them using a PCoA plot (Supplementary Figure S3, S4). Cows offered the HC diet had significantly more similar rumen bacterial communities (lower mean BC dissimilarities) compared to those offered the LC diet (*P* < 0.01). Similarly, when the diet was ignored, H-REI cows had significantly lower BC values than L-REI animals (*P* < 0.05). There were also significant differences (*P* < 0.01) in bacterial and archaeal BC values between H-REI and L-REI cows when both groups were offered the HC diet and for archaea but not bacteria when cows received the LC diet (Supplementary Figure S3, S4).

Differences between the diets were to a large extent explained by the higher abundance of *Bacteroidales* RF16 group, *Gastranaerophilales*, *Fibrobacter*, *Anaeroplasma*, *Absconditabacteriales* (SR1), and *Kiritimatiellae* WCHB1-41 in the LC diet. The rumen microbiota of cows offered the HC diet was significantly enriched in *Prevotellaceae* sp., *Clostridia* UCG-014, *Acetitomaculum*, and *Succinivibrionaceae* UCG-002 (Table 7). There was also a significantly higher abundance of *Methanobrevibacter* (*P* = 0.02) in L-REI cows offered the LC diet and more *Fibrobacter* when offered the HC diet compared to H-REI cows.

**Table 7:** The effects of residual energy intake (REI) and diet on rumen and fecal microbiota relative abundance (%).

Diet	H-REI^1^		L-REI^2^			*P* values		
	LC^3^	HC^4^	LC	HC	SEM	REI	Diet	REI × Diet
Rumen								
*Methanobrevibacter*	0.52	0.76	0.66	0.63	0.09	0.56	0.11	0.02
*Bacteroidales BS11 group*	0.42	0.17	0.33	0.17	0.06	0.71	0.01	0.58
*Bacteroidales RF16 group*	1.47	0.84	1.26	1.18	0.21	0.74	0.03	0.26
*Muribaculaceae*	1.84	2.48	2.28	2.11	0.21	0.86	0.09	0.04
*Prevotellaceae sp*	1.01	1.10	0.75	1.18	0.20	0.93	0.03	0.16
*Rikenellaceae RC9 group*	3.47	3.42	3.95	3.40	0.21	0.58	0.99	0.03
*Gastranaerophilales*	1.97	1.38	1.96	1.50	0.22	0.90	0.02	0.13
*Fibrobacter*	1.52	1.00	1.79	1.53	0.13	0.01	< 0.01	0.22
*Anaeroplasma*	0.81	0.56	0.66	0.56	0.08	0.85	< 0.01	0.14
*Erysipelatoclostridiaceae UCG-004*	0.36	0.20	0.31	0.20	0.05	0.63	0.03	0.48
*Erysipelotrichaceae sp*	0.08	0.14	0.09	0.04	0.03	0.39	0.86	0.03
*Christensenellaceae R-7 group*	2.58	2.83	2.79	2.07	0.26	0.15	0.68	0.02
*Clostridia UCG-014*	2.48	3.49	2.92	3.84	0.33	0.79	< 0.01	0.21
*Acetitomaculum*	0.90	1.61	1.02	1.35	0.15	0.86	< 0.01	0.03
*Lachnoclostridium*	0.12	0.26	0.18	0.17	0.03	0.33	0.06	0.01
*Lachnospiraceae ND3007 group*	0.23	0.36	0.13	0.30	0.06	0.17	0.04	0.12
*[Eubacterium] hallii group*	0.58	0.43	0.54	0.30	0.08	0.53	0.03	0.56
*[Eubacterium] ruminantium group*	1.36	1.75	1.51	1.28	0.16	0.36	0.23	0.02
*Ruminococcaceae sp*	0.20	0.81	0.66	1.10	0.14	0.03	< 0.01	0.11
*Ruminococcaceae CAG-352*	0.10	0.60	0.09	0.44	0.09	0.85	< 0.01	0.86
*Oscillospirales UCG-010*	0.37	0.21	0.43	0.26	0.06	0.27	< 0.01	0.28
*[Eubacterium]coprostanoli group*	0.58	0.44	0.61	0.50	0.09	0.26	0.02	0.23
*Family XIII AD3011 group*	0.37	0.26	0.27	0.25	0.04	0.64	0.03	0.19
*Anaerovibrio*	0.18	0.34	0.10	0.34	0.06	0.16	0.01	0.08
*Absconditabacteriales (SR1)*	0.92	0.82	1.06	0.84	0.12	0.88	0.02	0.15
*Rhodospirillales spp*	0.37	0.09	0.16	0.32	0.08	0.86	0.24	0.03
*Ruminobacter*	0.24	0.23	0.09	0.26	0.07	0.56	0.08	0.05
*Succinivibrionaceae UCG-002*	1.19	3.16	0.81	2.96	0.39	0.40	< 0.01	0.47
*Pyramidobacter*	0.14	0.11	0.17	0.11	0.02	0.16	0.02	0.74
*Synergistaceae sp*	0.27	0.38	0.31	0.19	0.09	0.16	0.86	0.04
*Kiritimatiellae WCHB1-41*	2.70	0.86	2.56	1.50	0.28	0.32	< 0.01	0.02
Feces								
*Bacteroides*	5.22	5.14	5.32	4.73	0.20	0.50	0.09	0.03
*Bacteroidales F082*	0.58	0.34	0.43	0.25	0.06	0.18	0.01	0.66
*Muribaculaceae*	0.93	0.93	0.64	1.27	0.13	0.48	0.03	0.02
*Alloprevotella*	1.07	0.71	0.97	0.68	0.14	0.70	0.03	0.57
*Prevotellaceae UCG-003*	2.45	1.86	2.29	2.49	0.17	0.05	0.11	0.79
*Anaeroplasma*	0.08	0.12	0.06	0.09	0.02	0.18	0.02	0.11
*Erysipelatoclostridium*	0.35	0.27	0.34	0.36	0.03	0.50	0.87	0.03
*[Anaerorhabdus] furcosa group*	0.22	0.17	0.13	0.26	0.03	0.45	0.07	0.00
*Bacilli RF39*	0.94	1.66	1.16	1.31	0.14	0.79	0.01	0.01
*Undefined Firmicutes*	1.39	1.04	1.29	1.16	0.14	0.99	0.03	0.64
*Clostridia UCG-014*	1.76	2.87	2.99	3.61	0.35	0.02	0.08	0.05
*Lachnospiraceae sp*	3.52	5.56	3.91	4.65	0.34	0.89	0.05	0.09
*Blautia*	0.24	0.36	0.17	0.41	0.05	0.22	0.00	0.17
*Dorea*	0.67	0.37	0.58	0.64	0.07	0.33	0.18	0.01
*[Eubacterium] hallii group*	0.35	0.10	0.27	0.18	0.05	0.78	0.01	0.21
*[Ruminococcus] gauvreauii group*	0.42	0.16	0.42	0.25	0.08	0.13	0.02	0.30
*Incertae Sedis*	0.19	0.29	0.25	0.3	0.03	0.75	0.02	0.77
*Oscillospirales UCG-010*	7.45	5.98	7.57	5.48	0.72	0.39	0.00	0.07
*Clostridioides*	0.53	0.44	0.44	0.29	0.06	0.07	0.07	0.03
*Spirochaetaceae GWE2-31–10*	0.11	0.33	0.22	0.11	0.03	0.61	0.25	0.01

Only significantly affected bacterial and archaeal genera are presented.

^1^High REI group of cows.

^2^Low REI group of cows.

^3^Containing a low (30% DM) proportion of concentrates in the total mixed ration.

^4^Containing a high (50% DM) proportion of concentrates in the total mixed ration.

### Fecal microbiota

Alpha diversity of fecal bacteria and archaea did not show significant differences between the groups in response to diets or REI of the animals (Supplementary Figure S5). Across the whole data set, fecal bacteria were dominated by Firmicutes and Bacteroidota, with Verrucomicrobiota, Spirochaetota, Patescibacteria, Proteobacteria, Actinobacteriota, and Fibrobacterota, among others, representing low abundance phyla. The ten most abundant genera that made up to 50% of all sequencing reads were affiliated with *Oscillospirales* UCG-010, *Rikenellaceae* RC9 gut group, *[Eubacterium] coprostanoligenes* group, *Bacteroides*, *Oscillospiraceae* UCG-005, *Lachnospiraceae* sp., *Alistipes*, *Christensenellaceae* R-7 group, *Clostridia* UCG-014, and *Bacteroidales* RF16 group. The fecal archaeal community was dominated by *Mbb. gottschalkii* and *Mbb. ruminantium* clades, *Methanocorpusculum* and *Methanosphaera* ISO3-F5 with *Mmc.* Group 3a at low abundance was observed in two samples; however, archaeal composition showed high between-animal variation even within the same group (Supplementary Figure S6).

There was no significant difference in BC dissimilarities because of grouping samples by diet, but fecal bacterial and archaeal communities were more similar (lower mean BC dissimilarities) in L-REI cows than in H-REI cows (*P* < 0.05) when diet was ignored, and animals were grouped based on REI. There were also significantly lower (*P* = 0.01) bacterial BC values in L-REI compared to H-REI cows when both groups were offered the LC diet (Supplementary Figure S7, S8).

Fecal samples of animals fed the HC diet had a higher abundance of *Muribaculaceae* sp., *Bacilli* RF39, and *Lachnospiraceae* sp., while those fed the LC diet had a higher abundance of *Alloprevotella* and *Oscillospirales* UCG-010 (*P* < 0.05). L-REI animals, irrespective of diet, had significantly more *Prevotellaceae* UCG-003 and *Clostridia* UCG-014 (*P* < 0.05) in feces than H-REI cows (Table 7).

### Predicted functions of the rumen and fecal microbiota

Based on the Kyoto Encyclopedia of Genes and Genomes database, a total of 9 level-1, 39 level-2 and 254 level-3 pathways were predicted in our study. We examined predicted functional differences in rumen and fecal microbiota as a response to HC vs LC diets and as an effect of animal REI. The results indicate that in the rumen, a total of 18 and in feces, a total of 31 bacterial level-3 predicted functions were differentially abundant between the HC and LC diets, with a tendency of more functional categories enriched in the LC diet in both the rumen and feces. However, after multiple test correction, none of these differences remained significant.

When cows received the HC diet, a total of 18 level-3 predicted functional categories in the rumen indicated differences in abundance between H-REI and L-REI animals. Of them, 12 categories were enriched in H-REI cows (Supplementary Figure S9A). In feces, there were 23 functional categories with a tendency to have abundance differences between the cow REI groups. Of them, 12 were enriched in H-REI and 11 in L-REI animals (Supplementary Figure S9B). Much fewer predicted functional differences between H-REI and L-REI cows were observed when animals received the LC diet (Supplementary Figure S10). None of the significant differences remained after multiple test correction.

## Discussion

The objective of this study was to assess differences in nutrient use efficiency, rumen fermentation, methane emissions, and rumen and fecal microbiota composition between multiparous dairy cows assigned to L-REI and H-REI groups based on REI determined earlier during their first lactation. The most notable differences between REI groups were higher methane yield (g/kg DM intake), lower rumen pH, and higher rumen VFA concentrations for L-REI cows. In addition, L-REI cows derived more energy and protein from tissues, as indicated by negative energy and N balances and higher milk urea concentrations. Contrary to our expectations, there were no differences between the REI groups in feed intake, nutrient digestibility, milk yield, milk composition, or milk production efficiency. As expected, a higher proportion of concentrates in the diet increased nutrient intake and milk yield for both REI groups without evident interactions between REI status and diet. The community structure of the rumen and fecal microbiota was associated with both diet and REI, but the diet effect was more pronounced.

In recent years, RFI or REI have been suggested and evaluated as estimates of feed efficiency in monogastrics and ruminants. Residual energy intake is the measured amount of ME intake (MJ/d) that is higher or lower than ME intake predicted using a regression model that includes energy sinks for ECM yield, maintenance, BW change, and pregnancy^13^. Therefore, REI and RFI are independent of the energy sinks included in the model. In theory, REI accounts for variation in diet ME concentration, which is not considered by RFI. It is well established that ME intake is not a linear function of DM intake, as fiber digestibility decreases with increases in feed intake level and concentration of nonstructural carbohydrates in the diet^21^. True ME intake may be overestimated if adverse interactions between energy-enriched concentrates and forages with high fiber concentrations are not properly accounted for by the models^21^. In practice, RFI is more readily determined and adopted than REI because calculation of ME intake requires frequent sampling and chemical analysis of feed ingredients.

As reviewed by Herd and Arthur^34^, variation in RFI is associated with the level of feed intake, nutrient digestion and metabolism, physical activity, and thermoregulation. In the current study, no differences in nutrient digestibility were observed between L-REI and H-REI cows. Studies on the variation in nutrient digestibility in dairy cows suggest that between-cow variation in nutrient digestibility is not the major component of RFI. Mehtiö et al.^35^ reported an SD of 12 g/kg for between-cow variation in OM digestibility using acid insoluble ash as an internal digestibility marker. Cabezas-Garcia et al.^36^ reported an SD of 10 g/kg for between-cow variation in OM digestibility determined mainly by total collection of feces. Nutrient digestibility is affected by feed intake such that increases in the level of DMI decrease rumen retention time and digestibility of nutrients, especially those with slow rates of digestion, such as NDF^21^. As animals with low RFI consume less DM compared to their counterparts with high RFI, they can digest slowly digestible nutrients more extensively than animals with higher intake levels^34,37^.

In the current study, L-REI cows produced more heat as a proportion of GE intake than H-REI cows. However, this effect was as much due to numerically smaller GE intake as it was due to numerically higher heat production (MJ/d, data not shown). As heat production was not measured directly but was predicted based on the equation suggested by Brouwer (see the Materials and Methods above)^15^, the higher proportional heat production in L-REI cows was mainly explained by numerically higher O_2_ consumption and CO_2_ emissions compared to H-REI cows. Higher O_2_ consumption and CO_2_ emissions relative to GE intake could potentially be explained by higher oxidation of nutrients derived from body tissues in L-REI cows. Heat production of lactating dairy cows arises from cellular metabolism, physical work of the digestive tract, and activities related to locomotion, eating and rumination. Studies on mitochondrial function in steers indicated differences in cellular level energy metabolism between feed-efficient and -inefficient animals^38^. Lean tissue accretion requires less energy than fat deposition, but lean tissue is metabolically more active and has higher maintenance energy consumption than fat tissue^39^. Owing to differences in body composition, there is substantial variation in maintenance energy requirements between breeds and individuals within breeds.

The energy balance measured in the current study was lower (− 37 MJ/d) for L-REI cows than for H-REI cows (− 17 MJ/d). Catabolism and oxidization of tissue energy increase CO_2_ emissions relative to feed intake. Higher CO_2_ emissions per unit of DMI or OM digested for L-REI cows could thus be attributed to the negative energy balance. In addition, L-REI cows excreted a greater proportion of N in milk and urine and had higher milk urea concentrations than H-REI cows. These effects were associated with lower N balance for L-REI (− 6 g/d) than H-REI cows (+ 19 g/d), although N balance in H-REI cows tended to be affected by the diet (P = 0.10 for the interaction) such that N balance was higher on the HC diet for H-REI cows. These findings suggest that L-REI cows catabolized tissue energy and protein to sustain higher milk energy output relative to ME intake (*P* = 0.08; Table 6) compared to H-REI cows.

The physical activity of dairy cows is not readily measured and is typically not included in RFI models. Olijhoek et al.^9^ estimated that physical activity accounted for 6 to 7% of the variation in the DMI of lactating Holstein and Jersey cows. The residual energy intake of cows recruited to this experiment was determined in a free stall barn that allowed free locomotion of cows. Under those conditions, higher physical activity would be dissipated as heat, resulting in higher REI than in less active counterparts. In the current study, heat production (Table 6) expressed as a proportion of GE intake was measured in respiration chambers that did not allow normal physical activity.

Methane emissions represent an energy sink typically not accounted for in RFI or REI models. Olijhoek et al.^9^ estimated that methane emissions accounted for 8.5 to 8.7% of the variation in DMI for first- and second-parity Holstein cows, respectively. In the current study, methane yield or methane energy as a proportion of energy intake were higher in L-REI cows than in H-REI cows. These differences were associated with lower rumen pH and higher VFA concentrations for L-REI cows, but there were no differences in rumen fermentation patterns between REI groups. Differences in rumen pH, VFA concentrations, and methane emissions could be related to variation in rumen liquid passage kinetics or volume. Greater rumen VFA concentrations and lower rumen pH for L-REI cows could be related to a slower rate of VFA absorption, slower rate of liquid passage, or smaller rumen liquid volume. As discussed by Ramin and Huhtanen^40^, increases in the ruminal passage rate decrease the rumen retention time of microbes and thereby increase microbial cell yields. As microbial cells represent a substantial hydrogen sink alternative to methane, a slower rumen passage rate could be associated with increases in methane emissions.

In the absence of differences between RFI groups in rumen pH or rumen VFA concentrations, Rius et al.^23^ observed smaller rumen volume, lower rumen liquid flow (L/h), and higher fractional dilution rate of rumen liquid for cows with negative RFI. In a study with low- and high-methane yield sheep, significant differences between groups were observed in mean particulate and liquid digesta retention times. Compared to high-methane yield sheep, low-methane yield sheep had shorter retention times of both particulate and liquid digesta, smaller loads of rumen particulate matter contents, and smaller rumen volumes^41^. These findings suggest that rumen dynamics may partly explain between-animal variation in methane emissions.

Consistent with the current observations, Olijhoek et al.^42^ reported a higher methane yield (L/kg DMI) for low RFI cows than for high RFI cows, an effect that was associated with increases in NDF digestibility. A positive relationship between methane emissions and diet digestibility is associated with longer rumen digesta retention time in high methane emitter cows compared with low emitters^36^. Another rumen retention time-associated variable that affects methane yield is the efficiency of microbial N synthesis. A shorter rumen retention time increases the efficiency of microbial N synthesis, thereby increasing microbial cells as an alternative sink for methane synthesis^36^. Owing to these mechanisms, methane yield is expected to be lower for cows that have shorter rumen retention times.

Residual feed intake is often measured using standard diets with a fixed proportion of concentrates^13^. However, compared to present-day practices, future dairy cow nutrition and management must consider various aspects of sustainability. Alleged competition for human-edible food is one such aspect that cannot be overlooked in livestock nutrition. Future dairy cow diets may consist of fewer human-edible ingredients (grains and pulses) and more byproducts and forages^6^. Therefore, feed efficiency should be evaluated on different diets to ascertain that there are no interactions between efficiency and diet composition. In the current study, the HC diet represented a typical diet for dairy cows in early lactation, whereas the LC diet represented a diet with a lower proportion of human-edible ingredients. Previous observations suggest that the mechanisms underlying RFI may be diet dependent. Potts et al.^37^ concluded that digestibility accounted for between 9 and 31% of the variation in RFI when cows were fed low-starch diets, but it did not play any role when cows were fed high-starch diets.

In general, diet effects on tissue energy deposition and mobilization should be considered cautiously in change-over studies with limited length of experimental periods. In the current study, no interactions between REI and diet were evident in feed intake, nutrient digestibility, milk production, rumen fermentation, or methane emissions. A higher proportion of concentrates in the diet increased DM intake (2.9 kg/d), the amount of OM digested (1.7 kg/d), and ECM yield (5.9 kg/d). Methane yield (g/kg DM intake) tended to decrease as a response to a higher proportion of concentrates in the diet. Increases in the amounts of concentrates often decrease methane yield (g/kg DM intake) by decreasing the proportion of fiber in digestible nutrients fermented in the rumen^9^. However, diet effects are dependent on the digestion physiology of the animal. This was substantiated by the findings of Olijhoek et al.^42^, who observed interactions between Holstein and Jersey cows in methane yield responses to low (32%) and high (61% of DM) proportion of concentrates in the diet. Methane yield in Holstein cows decreased by 27% with increases in concentrate proportion but only 14% in Jersey cows^42^. A meta-analysis of rumen fermentation patterns and stoichiometric prediction of rumen methane emissions in dairy cows^36^, suggested that diet was a much greater source of variation than between-cow variation. Consistent with this suggestion, in the current study, diet effects on methane emissions were more pronounced than those due to REI.

In this study, the microbiota composition was determined to evaluate the hypothesis that feed efficiency could be explained by the differences in rumen and/or fecal microbiota. The current results demonstrated that both diet and REI status were associated with rumen and fecal microbiota structural and functional differences, but the diet effect was more pronounced. Several genera were significantly affected by the diet; however, changes were detected only among the low abundance taxa in both the rumen and feces. The limited differences in microbial community composition between the diets could be explained by the fact that diets in this study did not represent extremes in forage to concentrate ratio and provided only small differences in terms of substrate composition available for microbial fermentation. Nevertheless, analysis of predicted microbial functions suggested that at the whole community ecosystem level, diets showed tendencies to induce differences in the bacterial functional processes in the rumen as well as in the large intestine. In both rumen and feces, a larger number of predicted functions differentiated the L-REI and H-REI groups, but only when animals received the HC diet. Diet composition may stimulate different processes related to microbiota and host animal roles in energy extraction. When cattle are offered high forage diets, feed intake controls the ruminal digestion and, to some level, microbial processes, but when animals are offered a diet with a higher proportion of easier digestible concentrates, metabolite-sensing and speed of VFA uptake by the host tissues may be more limiting factors for energy extraction^43^. Nevertheless, predictions of bacterial functions from 16S rRNA gene data are not very accurate and additional analyses including metagenome and metatranscriptome data would be required to elucidate microbiota functional differences between REI groups.

The lack of strong associations between individual microbial taxa and REI status in the current study agrees with the observations in a cohort of 87 Nordic Red dairy cows^44^. Efforts to identify individual microbial taxa as global biomarkers for feed efficiency may be challenging, as microbial community composition can be affected by diet^45^ and breed^46,47^. In the current study, L-REI cows fed the LC diet had a significantly higher abundance of *Methanobrevibacter* and more *Fibrobacter* in the rumen when offered the HC diet. In feces, L-REI animals had significantly more *Prevotellaceae* UCG-003 and *Clostridia* UCG-014 than H-REI cows. Welch et al.^48^ identified the families *Ruminococcaceae*, *Mogibacteriaceae,* and *Christensenellaceae* to be more abundant in the feces of efficient Angus steers, while efficient Holstein × Gyr crossbred dairy cattle raised under tropical conditions were associated with a higher proportion of several members from the families *Lachnospiraceae*, *Oscillospiraceae*, *Erysipelotrichaceae*, and *Carnobacteriaceae* in the rumen^11^. Noel et al.^47^ did not find microbial differences between RFI groups while studying rumen and fecal microbiota in Holstein and Jersey dairy cows. In addition, physiological differences related to beef vs dairy cattle rearing must be accounted for in understanding the role of the microbiome in interpreting the feed efficiency phenotype.

Instead of looking only at individual taxa differences, Shabat et al.^10^ and Tapio et al.^44^ demonstrated that looking at diversity and functional differences at the whole microbial ecosystem level is more informative. Feed-efficient Holstein Friesian cows offered a diet consisting of 30% roughage and 70% concentrate had reduced rumen microbial community diversity values, while inefficient Nordic Red dairy cows demonstrated enrichment of microbial metabolic pathways. In the current study, L-REI cows exhibited significantly lower bacterial richness but higher mean BC dissimilarities only when offered the HC diet. These findings are in line with previous observations. In feces, the opposite effect was observed, as L-REI cows fed the LC diet but not the HC diet had significantly lower mean BC dissimilarities and no differences in alpha diversity estimates compared to H-REI cows. On the predicted microbial functional level in the rumen, a tendency for functional enrichment in the H-REI cow group when animals received the HC diet was observed, while L-REI and H-REI cows appeared functionally more similar when fed the LC diet. The current observations indicate that the role of microbiota in explaining the feed efficiency phenotype could be diet dependent. In addition, not only the rumen but also the large intestine microbiota role needs to be assessed. Digesta that escapes ruminal degradation and enters the lower gut is composed of a higher percentage of fiber and other low-digestibility components. Subjected to further fermentation in the large intestine, it can contribute up to 8–9% of the total metabolizable energy intake of dairy cows and is affected by the diet composition, with the total VFA concentration in the cecum increasing with the higher proportion of grain in the diet^49^. Welch et al.^48^ compared the rumen, fecal and cecal microbiomes in feed-efficient and feed-inefficient Angus steers and demonstrated no differences in the rumen microbiome regarding RFI status but greater bacterial diversity in the ceca of the more efficient animals. Therefore, it is plausible to hypothesize that the energy extraction in the lower gut could be processed differently in efficient compared to inefficient animals, and therefore, both the rumen and lower intestine may contribute to defining the efficiency phenotype.

## Conclusions

Multiparous cows ranked as high REI and low REI during their 1st lactation had small differences in milk production efficiency, rumen fermentation pattern and nutrient digestibility. The current observations did not indicate any interactions between REI and the diet composition in feed intake, milk yield or composition, nutrient digestibility, or rumen fermentation. In contrast, rumen and fecal microbiota differences between REI groups were diet dependent. The cows deemed efficient based on negative REI derived more energy and protein from body tissues to support milk yield than cows with positive REI. These differences highlight the importance of REI determination over an extended period, preferably the entire first lactation, if not more. Higher methane emissions for L-REI cows suggested an inverse relationship between the efficiency of feed conversion and methane emissions. Overall, the current study identified several physiological mechanisms underlying the differences between high and low REI cows, but these could not be distinguished from one another. Therefore, further studies are needed to investigate the quantitative role of different physiological mechanisms underlying feed efficiency.

### Supplementary Information

Supplementary Information.

## Data Availability

The sequencing data generated and analyzed during the current study are available in the NCBI Sequence Read Archive under BioProject Accession PRJNA922943.
