# Supplementary material for: The effects of residual energy intake on nutrient use, methane emissions and microbial composition in dairy cows
Source: Sci Rep. 2024 Jan 5;14:613. doi: 10.1038/s41598-024-51300-7 (PMC10770142; doi:10.1038/s41598-024-51300-7)
Supplement: Supplementary file 1 — Supplementary Information. [file 41598_2024_51300_MOESM1_ESM.docx]

**The effects of residual energy intake on nutrient use, methane emissions and microbial composition in dairy cows**

S. Ahvenjärvi,^1^ A. R. Bayat,^1^ M. Toivanen,^2^ P. Mäntysaari,^1^ and I. Tapio^1^

**Supplementary Information**

**A Rumen bacteria**


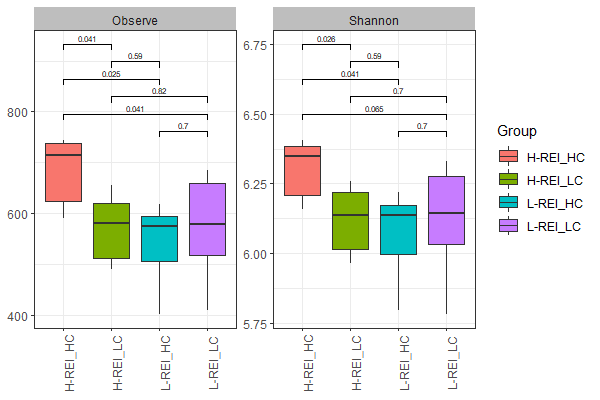


**B Rumen archaea**


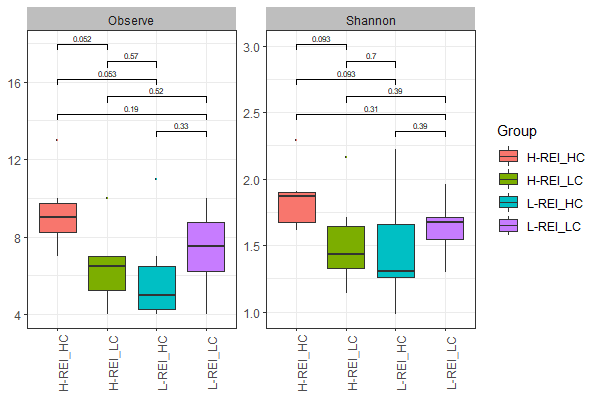


**Figure S1**. The rumen bacterial (**A**) and archaeal (**B**) alpha diversity expressed as observed number of ASV and Shannon diversity index. Significance of pairwise differences was estimated using non-parametric Wilcoxon test. Group definitions: H-REI – inefficient group, L-REI – efficient group receiving low concentrate (LC) or high concentrate (HC) diets.

**A Rumen bacteria**


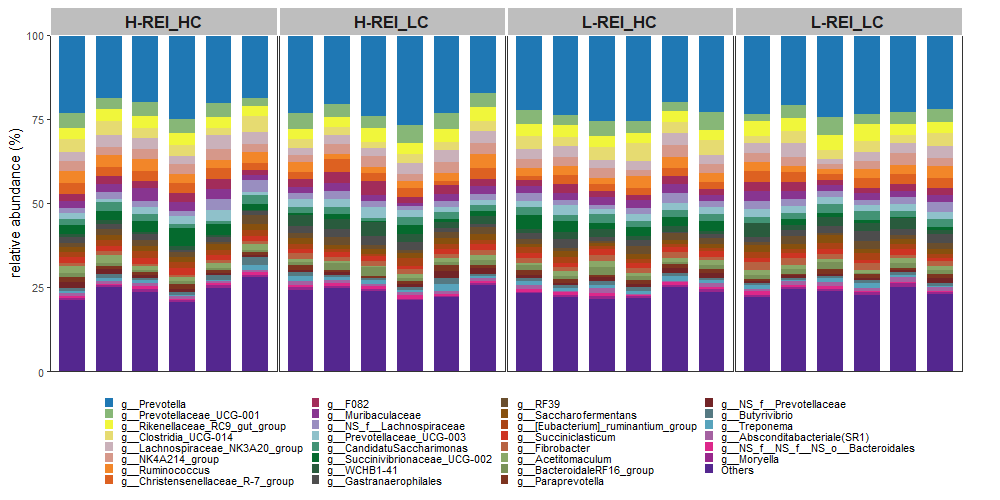


**B Rumen archaea**


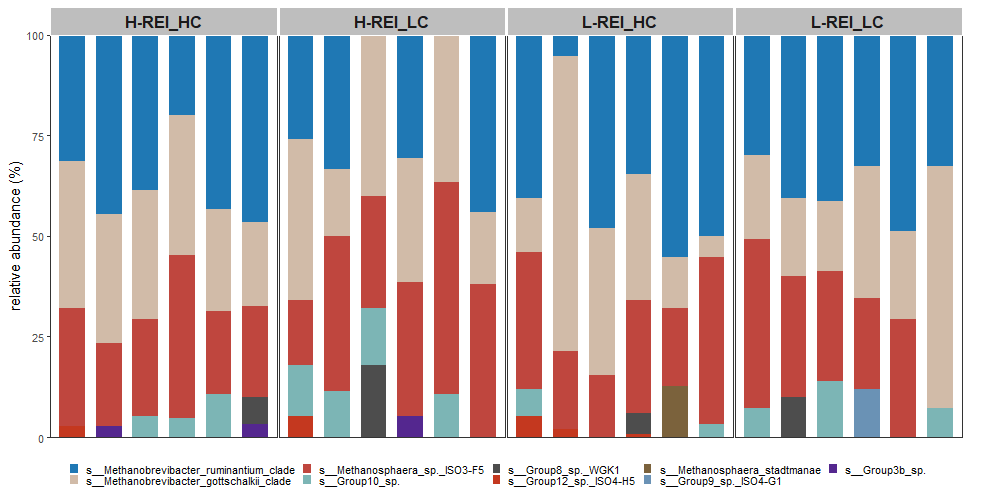


**Figure S2**. The rumen bacterial (**A**) composition at genus level and archaeal (**B**) composition at species level. Group definitions: H-REI – inefficient group, L-REI – efficient group receiving low concentrate (LC) or high concentrate (HC) diets.

**A Rumen bacteria**


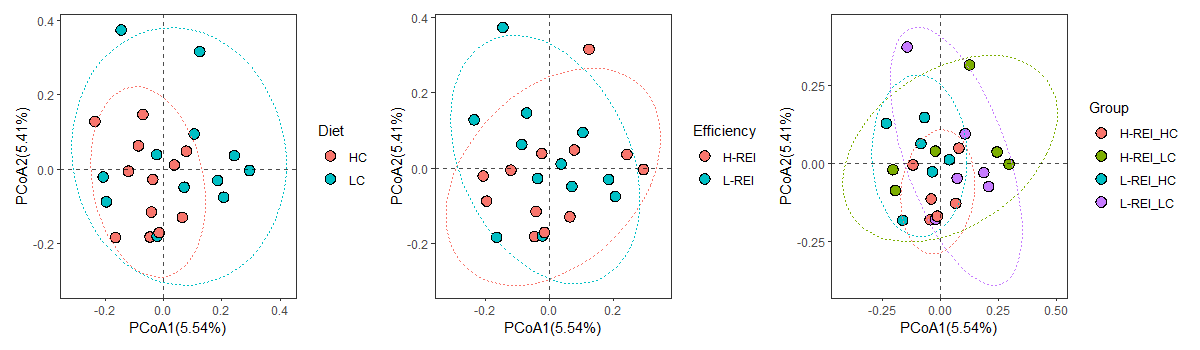


**B Rumen bacteria**


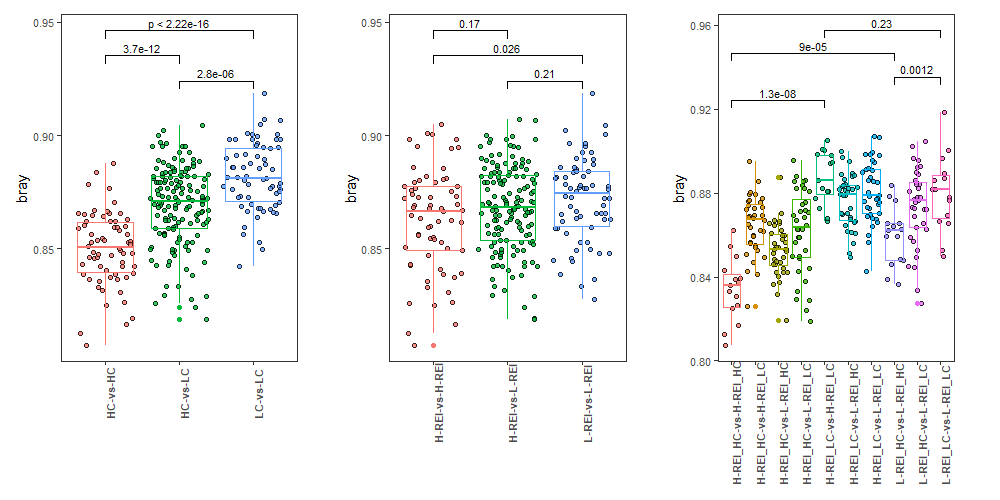


**Figure S3**. Rumen bacteria beta diversity. The principal coordinate analysis of Bray-Curtis dissimilarities (**A**) for samples grouped by diet (HC vs LC) (adonis test P = 0.02), REI group (H-REI vs L-REI) (P = 0.4) or REI/diet group (P = 0.05). (**B**) The boxplot of Bray-Curtis dissimilarities, evaluated between samples grouped by diet (HC vs LC), REI group (H-REI vs L-REI), or REI/diet group. Significance of pairwise differences was estimated using non-parametric Wilcoxon test.

Group definitions: H-REI – inefficient group, L-REI – efficient group receiving low concentrate (LC) or high concentrate (HC) diets.

**A Rumen archaea**


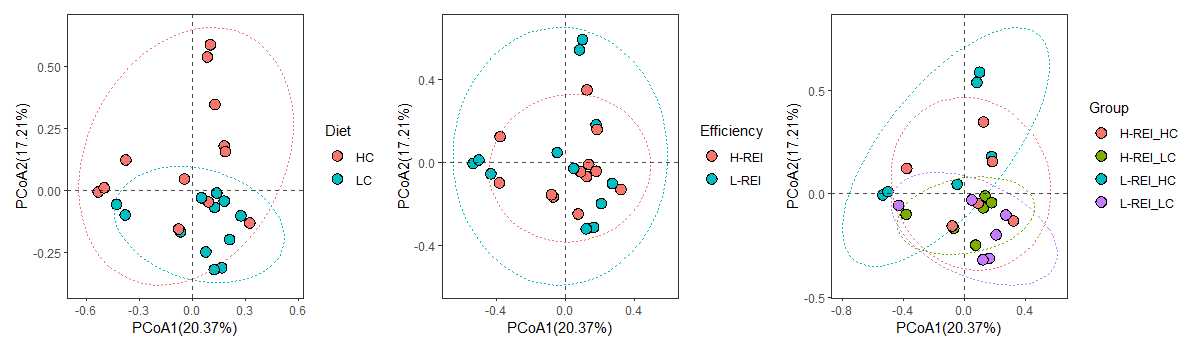


**B Rumen archaea**


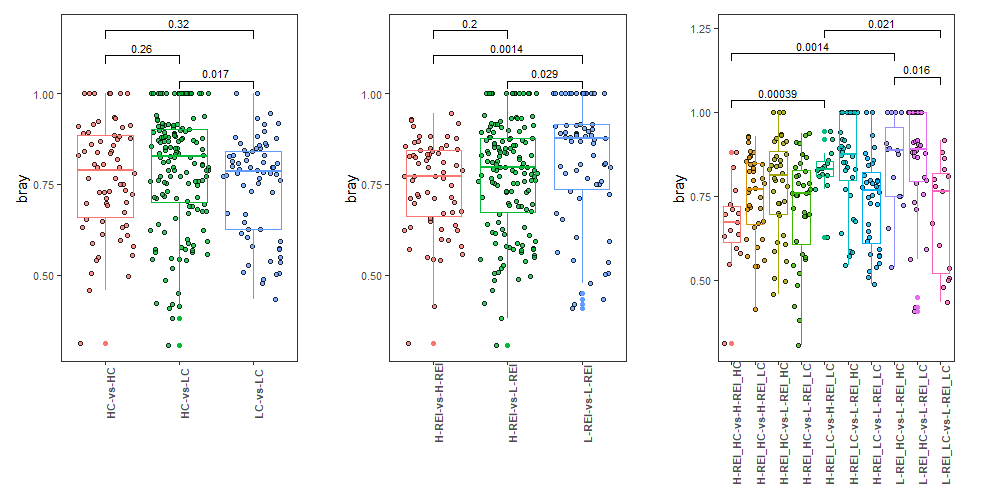


**Figure S4**. Rumen archaea beta diversity. The principal coordinate analysis of Bray-Curtis dissimilarities (**A**) for samples grouped by diet (HC vs LC) (adonis test P = 0.013), REI group (H-REI vs L-REI) (P = 0.78) or REI/diet group (P = 0.09). (**B**) The boxplot of Bray-Curtis dissimilarities, evaluated between samples grouped by diet (HC vs LC), REI group (H-REI vs L-REI), or REI/diet group. Significance of pairwise differences was estimated using non-parametric Wilcoxon test.

Group definitions: H-REI – inefficient group, L-REI – efficient group receiving low concentrate (LC) or high concentrate (HC) diets.

**A Fecal bacteria**


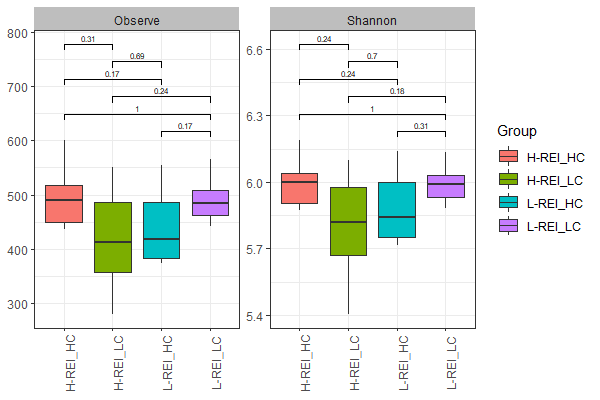


**B Fecal archaea**


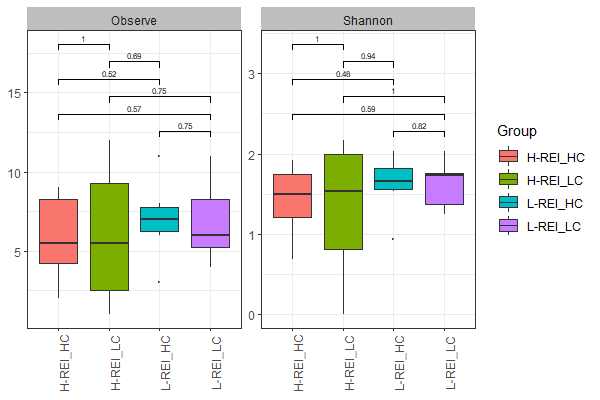


**Figure S5**. The fecal bacterial (**A**) and archaeal (**B**) alpha diversity expressed as observed number of ASV and Shannon diversity index. Significance of pairwise differences was estimated using non-parametric Wilcoxon test. Group definitions: H-REI – inefficient group, L-REI – efficient group receiving low concentrate (LC) or high concentrate (HC) diets.

**A Fecal bacteria**


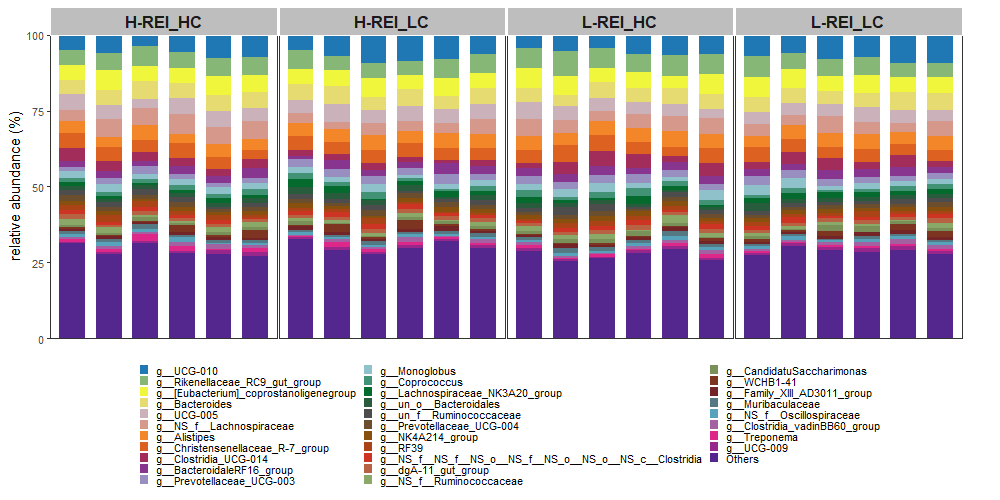


**B Fecal archaea**


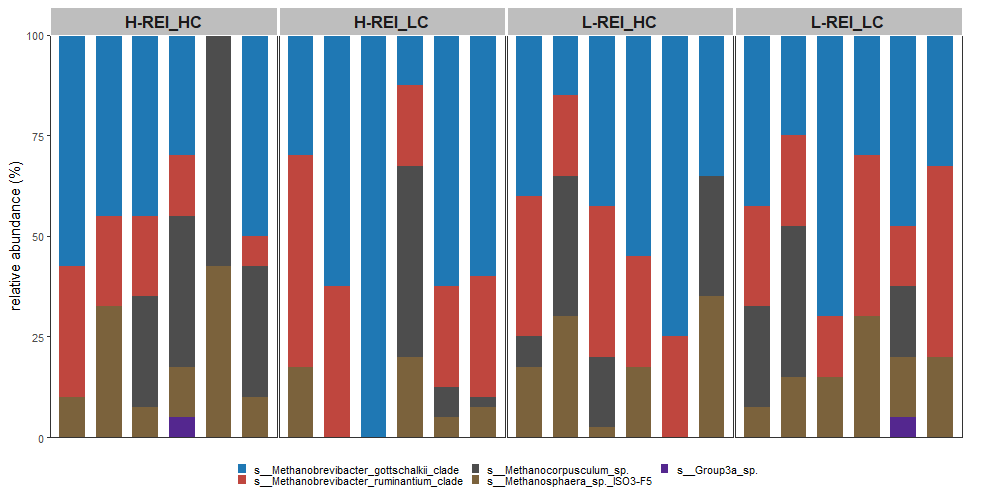


**Figure S6**. The fecal bacterial (**A**) composition at genus level and archaeal (**B**) composition at species level. Group definitions: H-REI – inefficient group, L-REI – efficient group receiving low concentrate (LC) or high concentrate (HC) diets.

**A Fecal bacteria**


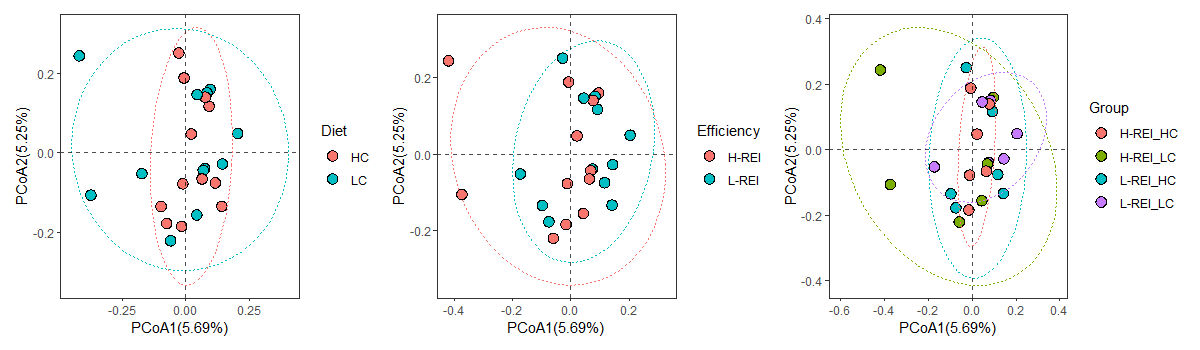


**B Fecal bacteria**


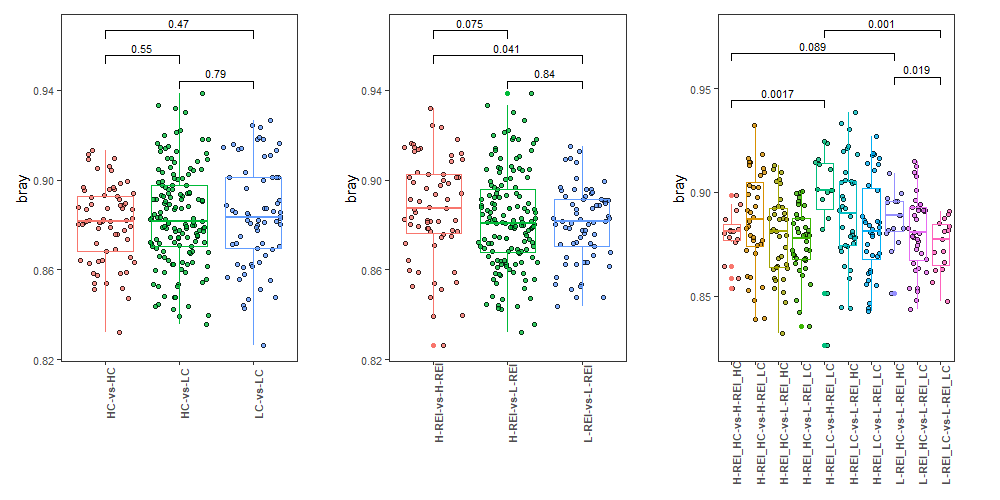


**Figure S7**. Fecal bacteria beta diversity. The principal coordinate analysis of Bray-Curtis dissimilarities (**A**) for samples grouped by diet (HC vs LC) (adonis test P = 0.23), REI group (H-REI vs L-REI) (P = 0.81) or REI/diet group (P = 0.74). (**B**) The boxplot of Bray-Curtis dissimilarities, evaluated between samples grouped by diet (HC vs LC), REI group (H-REI vs L-REI), or REI/diet group. Significance of pairwise differences was estimated using non-parametric Wilcoxon test.

Group definitions: H-REI – inefficient group, L-REI – efficient group receiving low concentrate (LC) or high concentrate (HC) diets.

**A Fecal archaea**


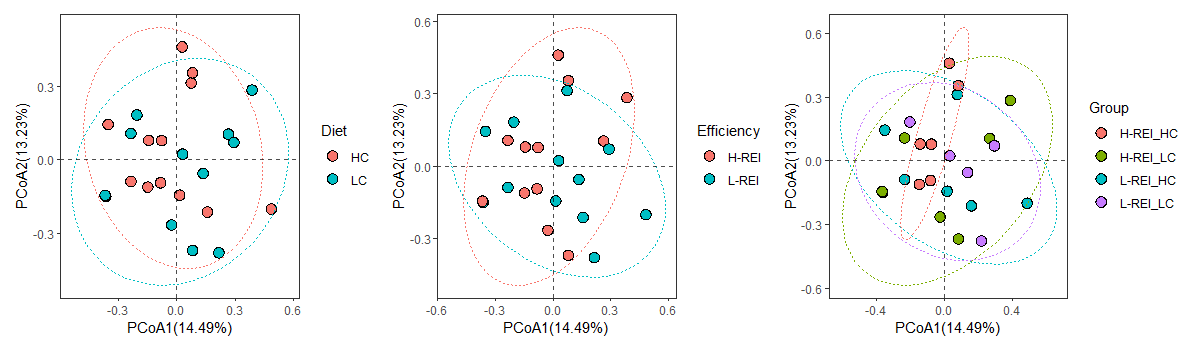


**B Fecal archaea**


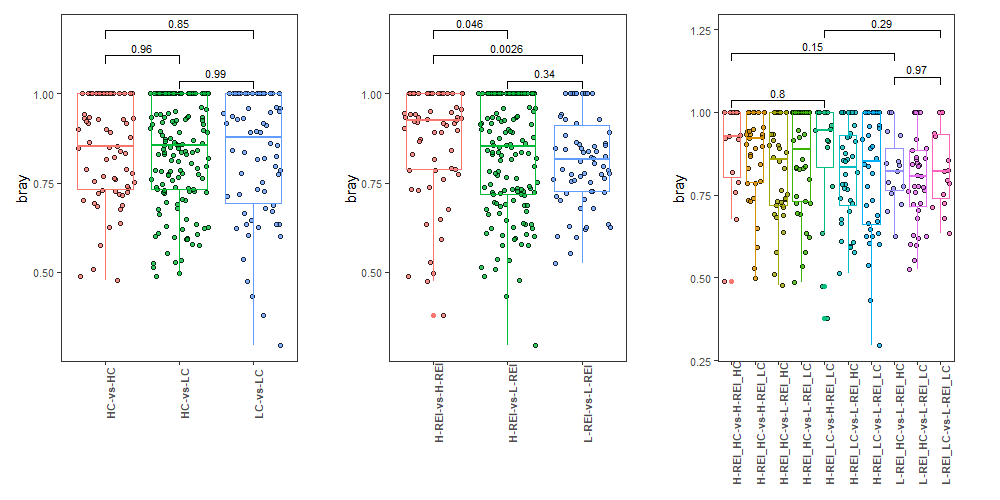


**Figure S8**. Fecal archaea beta diversity. The principal coordinate analysis of Bray-Curtis dissimilarities (**A**) for samples grouped by diet (HC vs LC) (adonis test P = 0.12), REI group (H-REI vs L-REI) (P = 0.89) or REI/diet group (P = 0.68). (**B**) The boxplot of Bray-Curtis dissimilarities, evaluated between samples grouped by diet (HC vs LC), REI group (H-REI vs L-REI), or REI/diet group. Significance of pairwise differences was estimated using non-parametric Wilcoxon test.

Group definitions: H-REI – inefficient group, L-REI – efficient group receiving low concentrate (LC) or high concentrate (HC) diets.

**A Rumen**


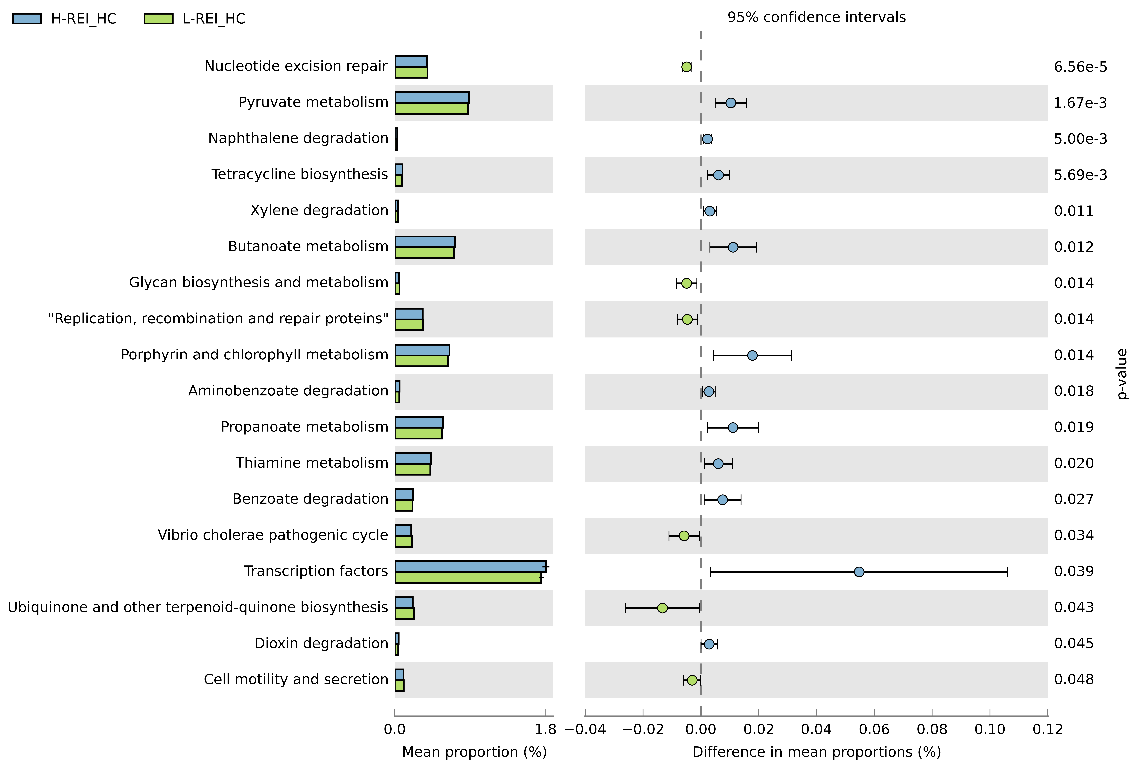


**B Feces**


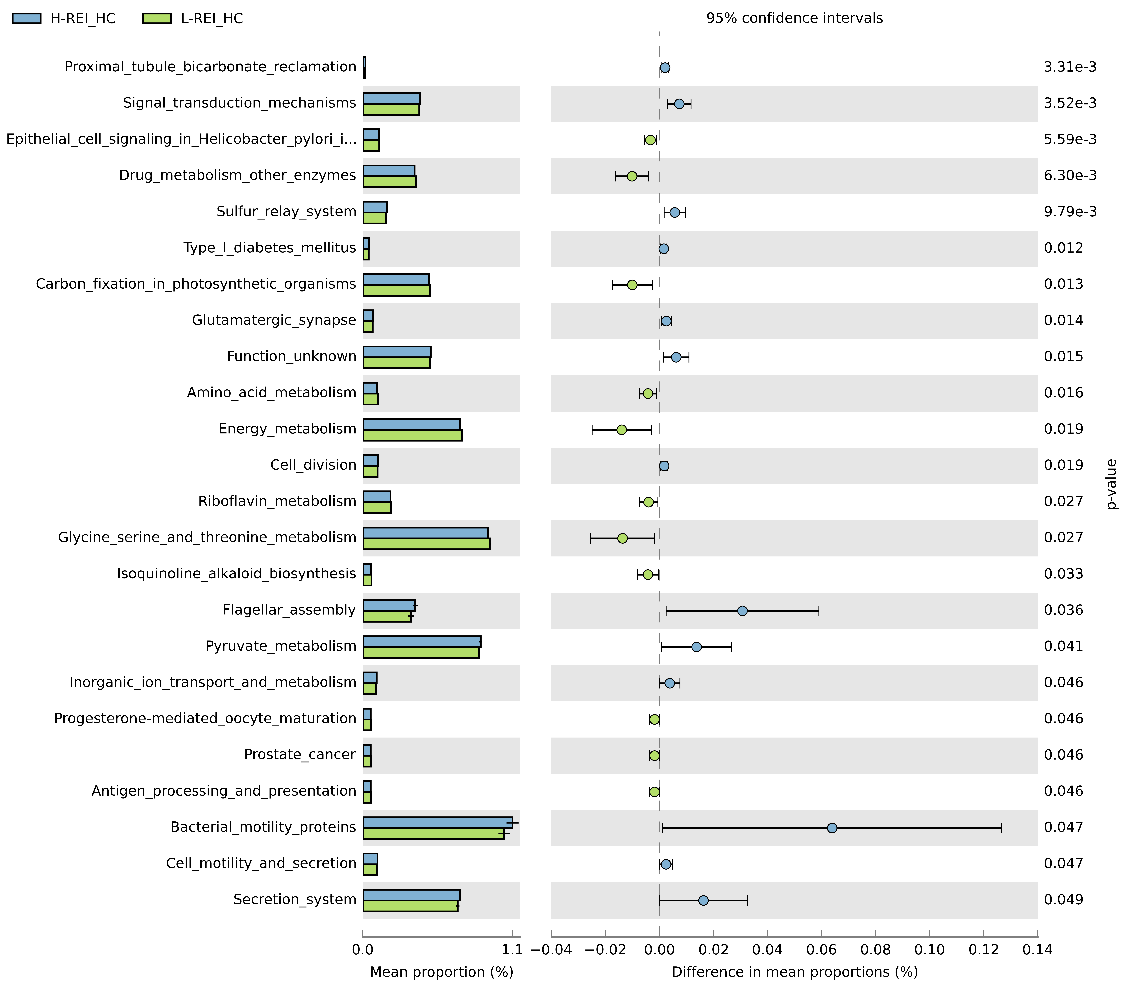


**Figure S9**. Differential predicted KEGG functions in (**A**) rumen and (**B**) feces between the H-REI and L-REI cows when animals received HC diet. Significance of pairwise differences was estimated using two-sided Welch’s t-test. After multiple test correction, no significant differences remained.

**A Rumen**


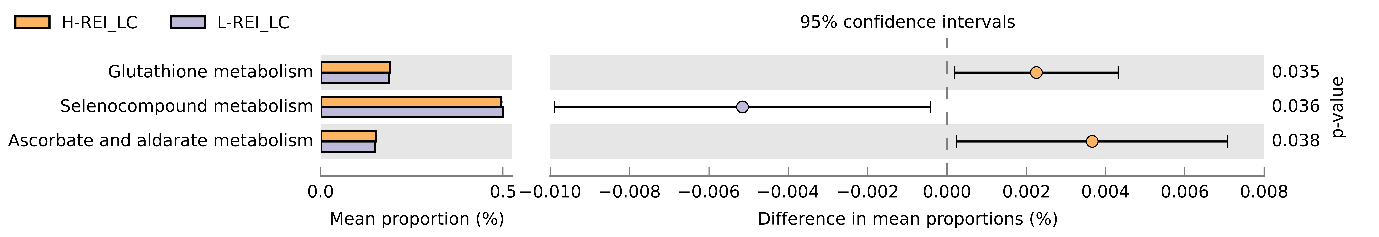


**B Feces**


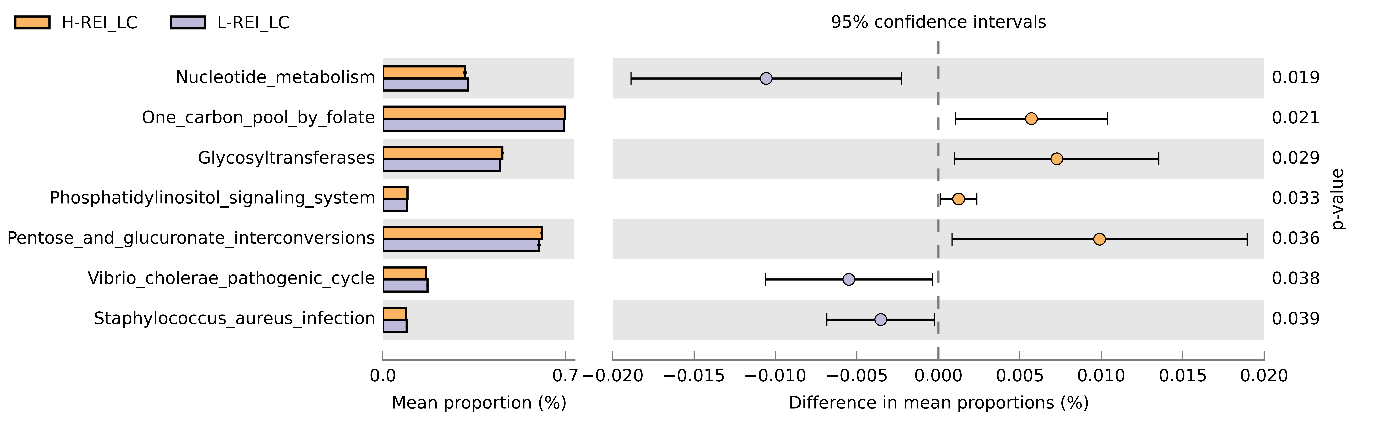


**Figure S10**. Differential predicted KEGG functions in (**A**) rumen and (**B**) feces between the H-REI and L-REI cows when animals received LC diet. Significance of pairwise differences was estimated using two-sided Welch’s t-test. After multiple test correction, no significant differences remained.
